# Assessment of accredited veterinary diagnostic laboratory use of breakpoints for canine and feline *Escherichia coli* infections in the United States and Canada

**DOI:** 10.3389/fvets.2023.1178522

**Published:** 2023-05-09

**Authors:** Brandon J. Durr, Larry D. Ballard, Angela D. Knight, Keun-Seok Seo, Vernon C. Langston, Alexis C. Thompson, Jacob M. Shivley, W. Cooper Brookshire

**Affiliations:** ^1^Department of Clinical Sciences, College of Veterinary Medicine, Mississippi State University, Starkville, MS, United States; ^2^Department of Pathobiology and Population Medicine, College of Veterinary Medicine, Mississippi State University, Starkville, MS, United States; ^3^Department of Comparative Biomedical Sciences, College of Veterinary Medicine, Mississippi State University, Starkville, MS, United States

**Keywords:** veterinary, antibiotic susceptibility testing, canine, feline, *E. coli*, breakpoint, antibiotic stewardship, ampicillin

## Abstract

The objective of this study was to assess the use of breakpoints in antibiotic susceptibility testing among veterinary diagnostic laboratories in the United States and Canada. An eight-question survey was conducted via phone and email to determine how often laboratories use breakpoints consistent with published guidelines in wounds, lower urinary tract infections and upper urinary tract infections (pyelonephritis) involving *Escherichia coli*, both in dogs and cats, for a total of 6 different hypothetical clinical scenarios. Nineteen veterinary diagnostic laboratories that perform antibiotic susceptibility testing on samples from dogs and cats in the United States or Canada and were accredited by the American Association of Veterinary Laboratory Diagnosticians (AAVLD) responded to the survey between January 15th and September 15th, 2022. The overall response rate of laboratories that were not excluded for known lack of dog and cat antibiotic susceptibility testing was 19 of 44 laboratories. Of the 17 respondent laboratories that reported using minimal inhibitory concentration breakpoints, only four laboratories used breakpoints consistent with published guidelines in all six clinical scenarios included in the survey. Our results suggest that there is clinically important variation in what breakpoints laboratories use to determine antibiotic susceptibility, which is of antibiotic stewardship and clinical relevance. Using breakpoints that are too high, too low, or inappropriately reporting “not interpreted” as the interpretive category may result in inappropriate use of antibiotics.

## 1. Introduction

Antibiotic susceptibility testing (AST) is commonly utilized by veterinarians to aid in clinical decision making about antibiotic therapy. The three primary components of broth dilution-based AST include bacterial species identification, measurement of minimal inhibitory concentration (MIC), and interpretive category determination. Alternatively, some laboratories utilize Kirby-Bauer zone diameter (ZD)-based AST, which includes bacterial species identification, measurement of zone of inhibition diameter, and interpretive category determination. To determine the interpretive category (i.e., susceptible, intermediate, resistant, or sometimes no interpretation is provided), the MIC or ZD is compared to a breakpoint (BP) based on patient species, infection location (i.e., body site), bacterial species, and antibiotic tested (1). In the United States, veterinary diagnostic laboratories typically utilize BPs set by the Clinical and Laboratory Standards Institute (CLSI), which are derived from data from a variety of sources including *in vitro* studies, pharmacokinetic-pharmacodynamic studies, human clinical studies, and veterinary clinical studies (2–4).

Successful utilization of AST in a clinical setting relies on appropriate completion of many steps by both the clinician and diagnostic laboratory. Clinicians must collect specimens appropriately, provide accurate patient and clinical information on submission forms, transport specimens appropriately, interpret diagnostic laboratory test results correctly, and ultimately use information gained to select the most appropriate course of treatment for their patient. Diagnostic laboratories must adhere to strict quality control protocols, maintain equipment, use appropriate and up-to-date breakpoints, determine MIC or zone of inhibition diameter, and provide antibiotic susceptibility interpretive categories consistent with published guidelines for each antibiotic tested (1, 5). Many veterinary diagnostic laboratories choose to become accredited by the American Association of Veterinary Laboratory Diagnosticians (AAVLD). This prestigious accreditation provides formal recognition of laboratory competence, quality, and adherence to industry standards (6).

The most recently published CLSI document at the time of data collection for this study (January 15, 2022–September 15, 2022) describing veterinary BPs included infection site specific (e.g., skin-soft-tissue or urine) MIC BPs for *E. coli* infections in dogs (ampicillin) and cats (amoxicillin-clavulanic acid; herein, amox-clav) (3). This document also contains a ZD BP for *E. coli* (amox-clav) urinary infections in cats, but it does not contain ZD BPs for skin-soft-tissue *E. coli* infections such as wounds or pyelonephritis in dogs or cats, or lower urinary tract infections in dogs (3). A newly updated CLSI document (CLSI VET01S ED6:2023) became available February 20, 2023, but the BPs and other information cited in this manuscript did not change with the update (2). However, the newly updated document does include new ZD and MIC BPs for feline *E. coli* urinary tract infections (UTI). Any reference in this manuscript, herein, to CLSI guidelines will refer to the document that was available at the time of data collection. It is widely recognized in the veterinary literature that UTIs involving skin-soft-tissue, such as pyelonephritis or prostatitis, should utilize skin-soft-tissue BPs in AST, rather than urine BPs (3, 5, 7, 8). While pyelonephritis is much less common than uncomplicated lower UTIs, it is nevertheless very important for clinicians and diagnostic laboratories to be familiar with diagnostic testing for UTIs that involve tissue. Regarding *E. coli* BPs for dogs and cats with infection sites that necessitate the use of skin-soft-tissue BPs, CLSI guidelines state “with the exception of isolates from UTIs, *E. coli* and other Enterobacterales should be reported as resistant to ampicillin, amoxicillin, and amox-clav because the drug concentrations achieved according to the dosage regimen used to establish BPs are not high enough to reach the therapeutic target” (3). Therefore, it is acceptable for laboratories to report all non-lower urinary tract *E. coli* isolates as resistant to amox and amox-clav, for cats and dogs, regardless of MIC. In some cases, laboratories may report the interpretive category as “not interpreted” or “no interpretation,” which is listed on the AST report as NI, or the interpretation cell is left blank. An interpretation of NI may be appropriate and consistent with published guidelines when no BP exists for a given bacterium, antibiotic, and patient species, or if the BP is outside of the laboratory’s MIC test range. The NI interpretation is inappropriate in cases where the laboratory has sufficient data to provide a S, I, or R interpretation. However, NI may be confusing or frustrating for practitioners who aren’t familiar with when NI is appropriate versus when it is inappropriate (1, 9). When clinicians evaluate interpretations categorized as NI in MIC testing and the MIC is > the highest test dilution, this means there was bacterial growth at the highest tested dilution and the bacterial isolate is likely resistant, depending on what MIC range was tested and particularly if an inappropriate MIC range was tested (an appropriate MIC range includes concentrations equivalent to achievable tissue antibiotic concentrations at the infection site, which coincide with BPs). If the MIC is ≤ the lowest test dilution tested, this means there was no growth in the lowest tested dilution and the bacterial isolate is likely susceptible, provided an appropriate MIC range was tested. For MIC values that fall between the lowest and highest concentrations tested, the clinician may wish to contact a clinical pharmacologist for advice on whether adequate tissue concentrations can be reached for that MIC. When clinicians encounter NI interpretations in ZD testing, extrapolation of estimation of bacterial susceptibility may be more difficult, as the zone of inhibition diameter is not typically included on the report provided to clinicians (1).

A clinically important knowledge gap exists in the veterinary literature describing the rate that AAVLD accredited veterinary diagnostic laboratories use BPs consistent with CLSI guidelines, regarding skin-soft-tissue vs. urinary tract infections in dogs and cats. The objective of this study was to assess the use of breakpoints in AST among veterinary diagnostic laboratories in the United States and Canada for six hypothetical clinical scenarios. The scenarios included AST for samples submitted from three different infection sites (wounds, pyelonephritis, and lower urinary tract) for both dogs and cats involving *E. coli*. For dogs, the antibiotic in the scenario was ampicillin, and for cats, the antibiotic was amox-clav.

## 2. Materials and methods

A brief eight-question survey was assembled. The survey was reviewed by the Mississippi State University Institutional Review Board and was determined to be “not human subjects research.” The study involved neither animal subjects nor any animal specific data, therefore Institutional Animal Care and Use Committee approval was not applicable. A list of 67 accredited veterinary microbiology laboratories in the United States and Canada was obtained from the publicly accessible AAVLD official website (10). Twenty three laboratories were excluded for stating that they do not perform AST on dog or cat samples on their website or stating this upon initial contact. The remaining 44 laboratories were contacted and provided a survey via telephone, email, or a combination of both between January 15th and September 15th, 2022. Initially, laboratories were first contacted by phone, but after virtually all laboratory representatives requested to be contacted via email, subsequent laboratories were contacted via email with follow up phone calls as needed for non-responders. The survey included the following eight questions:

1. When urine and aerobic culture samples are received in your lab from dogs and cats with *E. coli*, do you perform broth dilution MIC testing or ZD testing to determine antibiotic susceptibility?
2. Which MIC (Or ZD, if applicable) platform (e.g., system or hardware) do you use?
3. For wound culture samples submitted from dogs, what ampicillin BPs do you use for *E. coli*?
4. For urine culture samples submitted from dogs where the clinician indicates the lower urinary tract as the infection site in the case history, what ampicillin BPs do you use for *E. coli*?
5. For urine culture samples from dogs where the clinician indicates pyelonephritis as the infection site in the case history, what ampicillin BPs do you use for *E. coli*?
6. For wound culture samples submitted from cats, what amox-clav BPs do you use for *E. coli*?
7. For urine culture samples submitted from cats where the clinician indicates the lower urinary tract as the infection site in the case history, what amox-clav BPs do you use for *E. coli*?
8. For urine culture samples from cats where the clinician indicates pyelonephritis as the infection site in the case history, what amox-clav BPs do you use for *E. coli*?

Survey results were collected, anonymized, and organized into a spreadsheet (Excel, version 2302; Microsoft Corp.). For each hypothetical clinical scenario, reported BPs were recorded, along with a classification by the authors of incorrect or correct, based on concordance with CLSI veterinary specific BPs (3). Incorrect and correct classifications will herein be referred to as consistent with published guidelines or inconsistent with published guidelines. Any lab that reported all *E. coli* wound and pyelonephritis infections as resistant to ampicillin and amox-clav, or those that utilized susceptible, intermediate, or resistant interpretations based on the published CLSI skin-soft-tissue BPs, were categorized as using BPs consistent with published guidelines (3). The six hypothetical clinical scenarios in this study have a veterinary specific MIC BP in the latest CLSI guidelines, therefore any laboratory that reported NI when using MIC BPs for any of the hypothetical clinical scenarios were categorized as using BPs inconsistent with published guidelines (3). Each laboratory’s answers to questions 1 and 2 are provided (Table 1). Each laboratory’s answers to questions 3–5, if they reported MIC BPs, are provided (Table 2). Each laboratory’s answers to questions 6–8, if they reported MIC BPs, are provided (Table 3).

**Table 1:** Reported methods of antibiotic susceptibility determination (MIC or Kirby-Bauer ZD) and reporting system platform for each laboratory.

Laboratory	MIC or ZD	Platform
1	MIC primarily	Sensititre
2	MIC	Sensititre
3	MIC primarily	Sensititre
4	MIC	Sensititre
5	MIC	BIOMIC
6	MIC	BIOMIC
7	MIC	BIOMIC
8	MIC	Sensititre
9	MIC	Sensititre
10	MIC	Sensititre
11	MIC	BIOMIC
12	MIC	BIOMIC
13	MIC primarily	Sensititre
14	MIC	Sensititre
15	MIC primarily	Sensititre
16	MIC	Sensititre
17^*^	MIC	BIOMIC
17^*^	ZD	BIOMIC
18	ZD	(No answer)
19	ZD	BIOMIC

* Laboratory 17 reported both MIC and ZD breakpoints.

**Table 2:** Reference MIC breakpoints (3) (BP) and MIC BPs for dog clinical scenarios for labs that reported MIC BPs.

	MIC breakpoints, ampicillin, dog*, E. coli*									Wound			Urine—lower urinary tract			Urine—pyelonephritis			*S*	*I*	*R*	*S*	*I*	*R*	*S*	*I*	*R*
*Reference BPs**^a^*	≤0.25 or R	0.5 or R	≥1 or R	≤8		*>8*	≤0.25 or R	0.5 or R	≥1 or R
Lab 1	≤0.25	0.5	≥1	≤8			≤0.25	0.5	≥1
Lab 2	≤0.25	0.5	≥1	≤8			≤8		
Lab 3	≤0.25	0.5	≥1	≤8			≤8		
Lab 4	≤0.25	0.5	≥1	≤8			≤8		
Lab 5	≤0.25	0.5	≥1	≤8			≤8		
Lab 6	≤0.25	0.5	≥1	≤8			≤8		
Lab 7	R	R	R	≤8			≤8		
Lab 8	R	R	R	≤8			≤8		
Lab 9	R	R	R	≤8			≤8		
Lab 10	R	R	R	≤8			≤8		
Lab 11	R	R	R	≤8			R	R	R
Lab 12	R	R	R	≤8			R	R	R
Lab 13	R	R	R	≤8		≥16	R	R	R
Lab 14	NI	NI	NI	≤8	NI	NI	NI	NI	NI
Lab 15	≤1	NI or R^c^	NI or R^c^	≤8		≥16	≤8		≥16
Lab 16	≤1	NI or R^c^	NI or R^c^	≤1	NI or R^c^	NI or R^c^	≤1	NI or R^c^	NI or R^c^
Lab 17^b^	NI	NI	NI	≤8		>8	R	R	R

Blank cells indicate that the laboratory does not use I or R BPs, and it can be inferred that all isolates with an MIC > than the S BP are reported as R. Green = laboratories that utilized BPs consistent with published guidelines in all three clinical scenarios. MIC value units = μg per mL.

a If a lab reported all isolates resistant, regardless of MIC, the R is listed in the cell, rather than a BP.

b Laboratory 17 reported both MIC and Kirby-Bauer breakpoints.

c Cells with NI or R indicate that the laboratory reported sometimes reporting all isolates with an MIC >1 as R or NI.

**Table 3:** Reference MIC breakpoints (3) (BPs) and reported BPs for cat clinical scenarios for labs that reported MIC BPs.

	MIC breakpoints, amox-clav^b^, cat*, E. coli*									Wound			Urine—lower urinary tract			Urine—pyelonephritis			*S*	*I*	*R*	*S*	*I*	*R*	*S*	*I*	*R*
*Reference BPs**^a^*	≤0.25 or R	0.5 or R	≥1 or R	≤8		*>8*	≤0.25 or R	0.5 or R	≥1 or R
Lab 1	≤0.25	0.5	≥1	≤8	R	R	≤0.25	0.5	≥1
Lab 2	≤0.25	0.5	≥1	≤8			≤8		
Lab 3	≤0.25	0.5	≥1	≤8			≤8		
Lab 4	≤0.25	0.5	≥1	≤0.25	0.5	>1.0	≤0.25	0.5	≥1
Lab 5	≤0.25	0.5	≥1			≥8			>8
Lab 6	≤0.25	0.5	≥1	≤8			≤8		
Lab 7	R	R	R	≤8			≤8		
Lab 8	R	R	R	≤8			≤8		
Lab 9	R	R	R	≤8			≤8		
Lab 10	R	R	R	≤8			≤8		
Lab 11	R	R	R	≤8			R	R	R
Lab 12	R	R	R	≤8			R	R	R
Lab 13	R	R	R	≤8		≥16	R	R	R
Lab 14	NI	NI	NI	≤0.25	0.5	>1	NI	NI	NI
Lab 15	≤1	NI or R^d^	NI or R	≤8		≥16	≤8		≥16
Lab 16	≤1	NI or R^d^	NI or R	≤1	NI or R^d^	NI or R^d^	≤1	N or R^d^	NI or R^d^
Lab 17^c^	R	R	R	≤8			R	R	R

Blank cells indicate that the laboratory does not use BPs for the S, I or R interpretation, and it can be inferred that all isolates with an MIC > than the S BP are reported as R, or in the case of Lab 5, all isolates with an MIC < than the R BP are reported as susceptible. Green = laboratories that utilized BPs consistent with published guidelines in all three clinical scenarios. MIC value units = μg per mL.

a If a lab reported all isolates resistant, regardless of MIC, the R is listed in the cell, rather than a BP.

b Only the amoxicillin portion of the BP is provided.

c Laboratory 17 reported both MIC and Kirby-Bauer breakpoints.

d Cells with NI or R indicate that the laboratory reported sometimes reporting all isolates with an MIC >1 as R or NI. MIC value units = μg per mL.

Descriptive statistics were used to classify the laboratories that responded and summarize their responses. To determine if one platform was used correctly more often regardless of the clinical scenario, a linear mixed model (PROC MIXED, SAS version 9.4; SAS Institute Inc) was performed with the proportion of labs that correctly used BPs (dependent variable) between the two reported MIC platforms (Sensititre vs. BIOMIC) (independent variable) and clinical scenario as a random effect. An alpha of 0.05 was selected to indicate statistical significance. Assumptions of homoscedasticity and normality were evaluated through visual assessment of the residuals.

## 3. Results

Of the 44 laboratories provided a survey, three were excluded due to incomplete or unusable survey responses, and 23 did not complete the survey. In total, 19 laboratories completed the study and were included in the analysis. The overall response rate for laboratories not excluded for known lack of dog and cat testing was 19 of 44 laboratories. Of the 19 laboratories, 17 were associated with veterinary medical colleges, and two were associated with a state or provincial government diagnostic system. Of the 19 laboratories, 18 were in the United States, and one was in Canada. Seventeen of the 19 laboratories reported using MIC BPs, and three of the 19 laboratories reported ZD BPs. One laboratory reported both MIC and ZD BPs, therefore this respondent was included in both the MIC and ZD groups in the previous sentence. Of the 17 laboratories that reported MIC BPs, 11 reported that they utilize the Sensititre (Thermo Fisher Scientific) platform, and 6 reported that they utilize the BIOMIC (Giles Scientific) platform. Of the three laboratories that reported ZD BPs, two reported that they use BIOMIC and one did not answer the question.

Of the 17 laboratories that provided MIC BPs, only four used BPs in each of the six hypothetical clinical scenarios consistent with published guidelines. Of the 11 laboratories that reported using the Sensititre platform, two used BPs in each of the six hypothetical clinical scenarios consistent with published guidelines. Of the six laboratories that reported using the BIOMIC platform, two used BPs in each of the six hypothetical clinical scenarios consistent with published guidelines. When controlling for clinical scenario, laboratories using Sensititre reported MIC BPs consistent with published guidelines at a rate of 57.4% and laboratories using BIOMIC reported MIC BPs consistent with published guidelines at a rate of 74.9% (*p* = 0.009).

Correct use of MIC BPs is summarized for each of the clinical scenarios in Table 4. When evaluating each scenario individually, most laboratories (>75%) reported using MIC BPs consistent with published guidelines for the wound and lower urinary tract infection scenarios in dogs and cats. However, fewer laboratories (<40%) use MIC BPs consistent with published guidelines in the pyelonephritis scenario in dogs and cats. The BP issues resulting in inconsistency with published guidelines included inappropriate use of NI, using incorrect BP numerical values, or a combination of both. More than half of laboratories reported using urine BPs, rather than skin-soft-tissue BPs, for the pyelonephritis scenario in both dogs and cats, even though they were provided with infection site information necessitating the use of skin-soft-tissue BPs.

**Table 4:** Clinical scenarios and % of laboratories (*n* = 17) that reported breakpoints consistent with clinical guidelines.

Clinical scenario	Laboratories (%)
Dog wound	76.5
Dog UTI	88.2
Dog pyelonephritis	29.4
Cat wound	82.4
Cat UTI	76.5
Cat pyelonephritis	35.3

The CLSI guidelines available at the time of this study provided ZD BPs for only one of the six clinical scenarios (*E. coli* feline lower urinary tract infection with amox-clav). Each of the three laboratories that reported ZD BPs used BPs consistent with published guidelines for this hypothetical clinical scenario. The reported BPs for the other five hypothetical clinical scenarios appear to be extrapolated from either human or other veterinary BPs. One lab reported using NI for wounds in both dogs and cats, and this may be an appropriate use of NI, since there is not a published BP for this specific patient species, bacteria, infection site, and antibiotic.

## 4. Discussion

Incorrect utilization of MIC BPs, which the survey results suggest is happening commonly among AAVLD accredited laboratories, is of both clinical and antibiotic stewardship concern. In cases where too low of a BP was used many isolates will be reported as resistant, when in fact they should be reported as susceptible. For example, respondent laboratories 4 and 14 reported using an MIC BP of S ≤ 0.25 μg per mL for amox-clav in feline lower urinary tract *E. coli* infections, but published guidelines suggest using an MIC BP of S ≤ 8. Using this example where too low of a BP is used, any *E. coli* isolate with an amox-clav MIC of 0.5, 1, 2, 4, or 8 μg per mL will be incorrectly interpreted as resistant. This error is of clinical and antibiotic stewardship concern and would likely lead to unnecessary escalation of antibiotic therapy (e.g., switching to a different antibiotic). One possible explanation for this specific error made by laboratories is that this MIC BP (feline lower urinary tract infection, *E. coli* and amox-clav) is one of the most recently updated BPs by CLSI, and laboratory protocol updates may have lagged behind document updates (3). In cases where too high of a BP was used, many isolates will be reported as susceptible, when in fact they should be reported as resistant. For example, laboratories 2, 3, 4, 5, 6, 7, 8, 9, 10, and 14 reported using an MIC BP of S ≤ 8 μg per mL for ampicillin in canine pyelonephritis infections submitted as a urine culture, but published guidelines suggest using an MIC BP of S ≤ 0.25 or calling all isolates resistant. Using this example where too high of a BP is used, any *E. coli* isolate with an ampicillin MIC of 0.5, 1, 2, 4, or 8 μg per mL will be incorrectly interpreted as susceptible. This error is of clinical concern and would likely lead to inappropriate prescribing by the clinician who ordered the AST. In cases where the laboratory inappropriately uses NI, the clinician may make prescribing errors such as unnecessarily avoiding preferred antibiotics because of the NI.

Laboratories that reported using BIOMIC as their primary MIC platform used MIC BPs consistent with published guidelines in this study more than laboratories that reported using Sensititre. This difference between reported platform and use of MIC BPs consistent with published guidelines use may be explained by many factors, most of which were not measured in this study. Plausible explanations for the observed difference could be the frequency at which BPs are updated in the software, flexibility of software (e.g., software that permits laboratory personnel to select skin-soft-tissue breakpoints for urine samples), or compatibility of MIC platforms with other software, such as medical records systems. Additionally, many laboratories use a combination of equipment and software to perform MIC testing, therefore it is possible that classifying laboratories as either Sensititre or BIOMIC may be an oversimplification.

There was less variation between the three laboratories that reported ZD BPs. However, there were other issues noted when analyzing our results. Presumably due to the lack of available veterinary specific ZD BPs, most BPs were likely extrapolated from different veterinary infection sites or from human AST standards. While extrapolated BPs may be better than providing no interpretation, these results should be used with great caution owing to species and infection site differences regarding pharmacokinetic parameters (1). This survey did not measure how and if laboratories disclose on AST reports if BPs are extrapolated or if the laboratories include precautionary statements when extrapolated BPs are used.

There appears to be considerable variation in how laboratories handle urine samples submitted for infections that should utilize skin-soft-tissue BPs. In most cases of reported BP use inconsistent with published guidelines, urine BPs were utilized even though the lab was provided clinical history that necessitates the use of skin-soft-tissue BPs. This survey lacked the scope to fully understand how and why laboratories use BPs in this specific scenario, but, nevertheless, several potential solutions exist. Laboratories should consider simply updating protocols to allow the use of skin-soft-tissue BPs for urine samples when the clinical history necessitates. Alternatively, continuing education efforts could focus on improving clinician knowledge of BPs and improving sample submission for AST. For example, when a clinician suspects pyelonephritis, they should consider submitting the sample as a non-urine culture (i.e., aerobic culture and antibiotic susceptibility, rather than a urine culture) or including submission form language such as “please use skin-soft-tissue BPs” when submitting urine cultures. Urine culture submission forms should be amended to make it simple and required for clinicians to indicate the suspected infection location (e.g., uncomplicated lower UTI vs. pyelonephritis). For example, these forms could include a question such as “do you suspect a possible soft tissue infection? (e.g., pyelonephritis, prostatitis, etc.)”.

Current CLSI veterinary guidelines state that urine BPs should be used for amoxicillin and amox-clav for “UTIs” in dogs and cats caused by Enterobacterales. However, these same guidelines use more specific “uncomplicated UTIs” for cefazolin in the same dog clinical scenarios (3). Human CLSI guidelines describe urine BPs for “uncomplicated UTIs” (11). The European Committee on Antimicrobial Susceptibility Testing document states that urine BPs should be used for “uncomplicated UTIs only” (12). In future veterinary CLSI guidelines documents, more specific and standardized language specifying that urine BPs should be used for “only uncomplicated UTIs,” rather than using the non-specific “UTI” terminology, in all applicable antibiotics may improve BP usage among veterinary laboratories in the United States. Furthermore, additional urine and skin-soft-tissue specific ZD BPs should be developed for use in in future veterinary CLSI guidelines documents.

In future studies, BPs and MIC test ranges should be assessed for additional antibiotics, patient species, bacterial species, and infection sites. Submission forms and procedures should be studied as improvements in these areas could facilitate improved BP usage consistent with published guidelines. More information should be collected to better understand the association between MIC platform used and use of BPs consistent with published guidelines. The use of extrapolated BPs and how information about the limitations of extrapolated BPs are communicated to clinicians on AST reports should be studied. And finally, inappropriate use of NI should be studied. With a better understanding of when and why these problematic NI interpretations are used, future AST protocols and procedures could be improved to provide clinicians with more useful information for their patients.

## Data availability statement

The original contributions presented in the study are included in the article/supplementary material, further inquiries can be directed to the corresponding author.

## Ethics statement

Ethical review and approval was not required for the study on human participants in accordance with the local legislation and institutional requirements. Written informed consent from the participants was not required to participate in this study in accordance with the national legislation and the institutional requirements.

## Author contributions

BD, LB, AK, K-SS, VL, JS, and WB contributed to the conception and design of the study. BD conducted the survey and organized the database. AT performed the statistical analysis. BD and WB wrote the initial draft of the manuscript. All authors contributed to the article and approved the submitted version.

## Conflict of interest

The authors declare that the research was conducted in the absence of any commercial or financial relationships that could be construed as a potential conflict of interest.

## Publisher’s note

All claims expressed in this article are solely those of the authors and do not necessarily represent those of their affiliated organizations, or those of the publisher, the editors and the reviewers. Any product that may be evaluated in this article, or claim that may be made by its manufacturer, is not guaranteed or endorsed by the publisher.
